# Online adaptive radiotherapy for bladder cancer using a simultaneous integrated boost and fiducial markers

**DOI:** 10.1186/s13014-023-02348-8

**Published:** 2023-10-06

**Authors:** Sana Azzarouali, Karin Goudschaal, Jorrit Visser, Maarten Hulshof, Marjan Admiraal, Niek van Wieringen, Jakko Nieuwenhuijzen, Jan Wiersma, Laurien Daniëls, Duncan den Boer, Arjan Bel

**Affiliations:** 1 Radiation Oncology, Amsterdam UMC location Vrije Universiteit Amsterdam, De Boelelaan 1117, Amsterdam, The Netherlands; 2 Cancer Center Amsterdam, Cancer Therapy, Treatment and quality of life, Amsterdam, The Netherlands; 3 Radiation Oncology, Amsterdam UMC location University of Amsterdam, Meibergdreef 9, Amsterdam, The Netherlands; 4 Amsterdam UMC location Vrije Universiteit Amsterdam, De Boelelaan 1117, Urology, Amsterdam, The Netherlands

**Keywords:** Online adaptive radiotherapy, Bladder cancer, Fiducial markers, Reoptimization, Artificial intelligence, Radiotherapy, CBCT, Focal boost, Cone Beam CT

## Abstract

**Purpose:**

The aim was to assess the feasibility of online adaptive radiotherapy (oART) for bladder cancer using a focal boost by focusing on the quality of the online treatment plan and automatic target delineation, duration of the workflow and performance in the presence of fiducial markers for tumor bed localization.

**Methods:**

Fifteen patients with muscle invasive bladder cancer received daily oART with Cone Beam CT (CBCT), artificial intelligence (AI)-assisted automatic delineation of the daily anatomy and online plan reoptimization. The bladder and pelvic lymph nodes received a total dose of 40 Gy in 20 fractions, the tumor received an additional simultaneously integrated boost (SIB) of 15 Gy. The dose distribution of the reference plan was calculated for the daily anatomy, i.e. the scheduled plan. Simultaneously, a reoptimization of the plan was performed i.e. the adaptive plan. The target coverage and V_95%_ outside the target were evaluated for both plans. The need for manual adjustments of the GTV delineation, the duration of the workflow and the influence of fiducial markers were assessed.

**Results:**

All 300 adaptive plans met the requirement of the CTV-coverage V_95%_≥98% for both the boost (55 Gy) and elective volume (40 Gy). For the scheduled plans the CTV-coverage was 53.5% and 98.5%, respectively. Significantly less tissue outside the targets received 55 Gy in case of the adaptive plans as compared to the scheduled plans. Manual corrections of the GTV were performed in 67% of the sessions. In 96% of these corrections the GTV was enlarged and resulted in a median improvement of 1% for the target coverage. The median on-couch time was 22 min. A third of the session time consisted of reoptimization of the treatment plan. Fiducial markers were visible on the CBCTs and aided the tumor localization.

**Conclusions:**

AI-driven CBCT-guided oART aided by fiducial markers is feasible for bladder cancer radiotherapy treatment including a SIB. The quality of the adaptive plans met the clinical requirements and fiducial markers were visible enabling consistent daily tumor localization. Improved automatic delineation to lower the need for manual corrections and faster reoptimization would result in shorter session time.

**Supplementary Information:**

The online version contains supplementary material available at 10.1186/s13014-023-02348-8.

## Introduction

Bladder cancer is the 9th most diagnosed cancer worldwide and approximately 1 out of 5 patients develop muscle invasive bladder cancer reducing the 5-year survival rate to 50% [1, 2]. Cystectomy is considered the standard treatment of muscle invasive bladder cancer and combining the surgery with radiotherapy (RT) has shown to improve the outcome [3]. However, the procedure can have significant side effects [4]. Combining RT with chemotherapy and transurethral resection of bladder tumor (TURBT) has shown to be an effective alternative while preserving the bladder [5–10].

The main challenge for treating this anatomical site with radiotherapy is the variable bladder size, shape and position. These variations occur both between (interfraction) and during (intrafraction) RT fractions [11]. Even though drinking instructions typically aim to limit the intrafraction variations, the extent remains unpredictable [12–15]. Interfraction bladder deformation can be accounted for with the application of appropriate (larger) margins, library of plans (LoP) or adaptive reoptimization of the plan. The latter two lead to smaller irradiated volumes than the former [16, 17]. For the LoP approach, different patient-specific treatment plans are made based on different bladder volumes and the best fitting plan is selected for each fraction. An adaptive procedure with plan reoptimization during each treatment session utilizes anatomical information from daily images as acquired with Cone Beam CT (CBCT) or magnetic resonance imaging (MRI) on the linac [18–20]. Preclinical studies of these two strategies have shown that daily reoptimization is superior compared to LoP with less integral dose and dose to organs-at-risk (OAR) [21, 22].

Using a focal boost for bladder RT has been shown to be feasible and could further reduce the toxicity [10, 23, 24]. Several studies have shown feasibility of giving such a focal boost as a simultaneous integrated boost (SIB) to the tumor bed [10, 23]. Delivery of a SIB can be complicated by the reduced visibility of the remaining tumor volume when TURBT is performed prior to RT [19]. However, implantation of fiducial markers has shown to facilitate gross tumor volume (GTV) localization and delineation [25, 26]. Liquid fiducial markers are known for their high density which is favorable for (CB)CT scans and the feasibility considering the visibility, stability and safety has been demonstrated [26].

To allow for daily plan reoptimization, different online adaptive radiotherapy (oART) techniques have been developed [19]. The techniques acquire an image of the daily anatomy at the beginning of each treatment fraction. These images are used as input for the reoptimization of the treatment plan. One of these techniques consists of the integration of MRI with a linear accelerator (linac). The modality offers high soft tissue contrast but leads to a relatively long treatment time which is a drawback when considering intrafractional bladder filling, patient comfort and additional work load [18]. A novel concept has been developed, integrating a linac, CBCT and artificial intelligence (AI)-driven software for automatic organ delineation and plan reoptimization [20]. The result of these developments is that on board CBCT and MRI are increasingly used modalities for oART. The median on-couch time (time from first image acquisition to the end of RT delivery) for oART with plan reoptimization using MRI was reported as 39 min [18]. For CBCT-guided oART the delineation of the daily anatomy until treatment plan selection took 12 min, but the total on-couch time, including RT delivery, has so far not been reported in literature [27, 28]. Several studies have shown bladder oART to be feasible and applicable with CBCT and the image quality to be sufficient to apply automatic bladder segmentation resulting in similar outcomes as manual delineation [29–31]. Recent studies on CBCT-guided oART show the technique to be feasible for whole bladder irradiation without a focal boost [27, 28]. So far no studies have evaluated the performance of CBCT-guided oART for muscle invasive bladder cancer including a focal boost and fiducial markers for tumor localization.

The aim of this study was to prospectively examine the feasibility of a CBCT-guided oART workflow for bladder radiotherapy using fiducial markers and a SIB. We will evaluate the automatic target delineation, quality of the online treatment plan, performance with fiducial markers, the duration of main steps of the workflow and the total on-couch time.

## Methods

### Patient characteristics

Fifteen patients with muscle invasive bladder cancer were treated between April 2021 and December 2022 on a ring-based linac integrated with a CBCT and software platform for both treatment planning and delivery (Ethos Therapy™, version 1.1, Varian a Siemens Healthineers Company, USA). The patients’ mean age was 68 years and the number of males and females was 11 and 4, respectively (see Additional file 1). In 20 fractions the bladder, urethra and first pelvic lymph nodes (elective volume) of each patient received a total dose of 40 Gy combined with a SIB of an additional 15 Gy to the tumor bed resulting in a total of 300 delivered fractions. Radiotherapy was combined with chemotherapy (Mitomycin-c/ Capecitabine).

### Pretreatment

Prior to acquiring the planning CT, liquid fiducial markers (BioXmark, Nanovi A/S, Denmark or Lipiodol) were injected by the urologist at the borders of the tumor bed. These markers aid in target localization for both the pretreatment on CT and the online fractions on CBCT. Fiducials were placed in an outpatient setting with rigid cystoscopy in women and flexible cystoscopy in men, after histological confirmation of the bladder tumor by TURBT. The fiducials were placed submucosally with a margin of 0–5 mm around the (remnant) tumor or around the resection scar (for simplicity we will refer to both as “GTV”). Preferably 3–5 dots of 0.1–0.2 cm^3^ were placed. Within 6 weeks after TURBT, each patient was asked to drink 0.3 L of water after voiding the bladder and subsequently refrain from drinking 1.5 h prior to CT acquisition (Discovery CT, GE Medical Systems). The CT was made in supine patient position with the arms on the chest and a knee support. To mimic the situation during the online adaptive procedure, after 15 min a second CT was acquired to estimate the intrafractional bladder filling. The first acquired planning CT (pCT) was used to delineate the GTV, the clinical target volume (CTV) of both the first pelvic lymph nodes (obturator, internal iliac and hypogastric lymph nodes and external lymph nodes (perivesical until lower part of sacroiliac joint)) and urethra (men: 2 cm proximal, women: 1 cm proximal) and OARs (bladder, rectum, bowel bag, sigmoid, left and right femur head), see also Additional file 2. If the GTV was in the cranial part of the bladder, the small bowel was also delineated. A GTV-CTV_SIB_ and CTV_SIB_-PTV_SIB_ margin of 5 mm were used. The CTV_elective_ consisted of the urethra, the pelvic lymph nodes and the bladder. The urethra and pelvic lymph nodes had a CTV_elective_ to PTV_elective_ margin of 5–7 mm.

The second CT scan was used to generate patient specific margins, to account for intrafraction bladder filling. Firstly, the bladder delineated on the first scan was expanded 5 mm in all directions. If the bladder on the second CT was completely encompassed by this expansion a uniform PTV_elective_ margin of 7 mm was used. In any direction where the uniform 5 mm expansion did not encompass the bladder, the necessary expansion to encompass the bladder was determined. This necessary expansion was then increased by 50% to account for the assumption that on-couch time during the online adaptive procedure is currently longer than the time interval of 15 min between the two CTs. With the use of the above described target and OAR volumes a reference plan was generated by using the automatic treatment planning system (TPS) of the Ethos, which utilizes prioritized clinical goals for optimization of the final dose distribution (3 arc VMAT, 6MV FFF). The template used for these clinical goals is given in the additional files (see Additional file 3). All treatment plans were normalized to 98% of the volume of PTV_SIB_ receiving 95% of the prescribed dose of 55 Gy.

### Online adaptive workflow

**Fig. 1:**
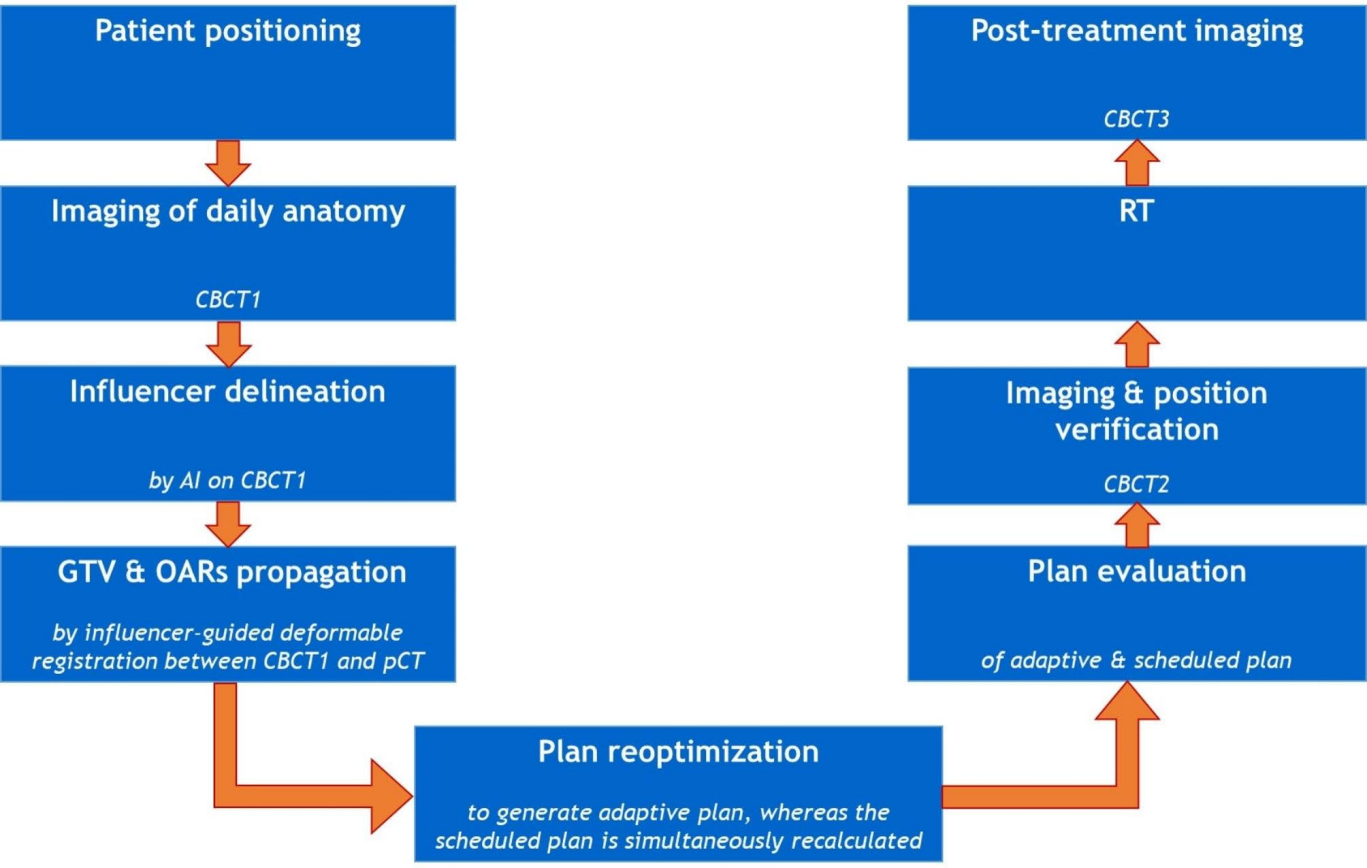
AI-driven CBCT-guided oART workflow

The online adaptive treatment sessions were run by a team of two radiation therapists (RTT). A physician and medical physics expert were either present in the operating room or reachable on call. At the start of each fraction a CBCT (CBCT1) was acquired. The bladder and rectum were automatically delineated by the software, using a convolution neural network, allowing for manual correction after presentation [20]. These delineations (so-called influencers) and a deformable registration of the planning CT to the CBCT, resulting in a synthetic CT (sCT), were used to propagate a structure set based on the anatomy of the day. Manual corrections to the target structure and OARs were performed by the RTT/physician if necessary. The dose distribution of the reference plan was calculated on the anatomy of the day using the new structures and the sCT, resulting in the so-called scheduled plan. At the same time, an adaptive plan was generated by running a new optimization. For both the scheduled and adaptive plan, an independent dose calculation was performed for plan QA (Mobius, Varian a Siemens Healthineers Company, USA). Subsequently, one of the two plans was selected for treatment (i.e. adaptive or scheduled plan) based on the clinical goals, regions of high dose (> 107% of the prescribed dose outside target volume) and isodose lines (target coverage and dose conformity of 95% and 107% of the prescribed dose). Prior to delivery, a second CBCT (CBCT2) was acquired for position verification, which consisted of a bone match and a check of whether the GTV was still encompassed by PTV_SIB_. If this was not the case, a couch shift was performed if the required shift was larger than 1 mm in one or more directions. To evaluate whether the elective and boost targets were inside the PTV margins during the actual irradiation, a post-treatment CBCT (CBCT3) was acquired (see also Fig. 1 for an illustration of the workflow). If the PTV margins were not adequate at the end of the session, the physician was consulted and the margins were adjusted in the TPS for the subsequent sessions.

### Workflow evaluation and statistical analysis

To evaluate the CBCT-guided oART workflow, the analysis was focused on the quality and consistency of its online treatment plans, accuracy of its target propagation, duration of the online sessions and the capability of the system with respect to handling fiducial markers. Due to the fact that introducing a technique in the clinic usually comes with a learning curve, the first five patients were considered as a training group. To test this assumption, the workflow performed on the training group was compared with the workflow performed on patient six until patient 15 (the steady group).

The quality and consistency of the scheduled and adaptive plans were analyzed with help of home built software (Matlab R2021a, Mathworks) by assessing the target coverage, conformity index (CI), homogeneity index (HI) and volume of healthy tissue outside the target receiving high dose (55 Gy/40 Gy) [32, 33]. To make the conformity comparable with previous studies a conversion of the CI was performed (CI_RTOG_) [34]. The target coverage is given by the percentage of the volume of the target receiving at least 95% of the prescribed dose (V_95%_). The clinical requirement for the target coverage of the PTV and CTV was a minimum of 98% for V_95%_.

The healthy tissue outside the target receiving high dose (> 55 Gy for tissue outside the GTV and > 40 Gy for tissue outside the elective volume) is given by

$${V}_{95\%, out}= {V}_{95\%, Body}- {V}_{95\%}$$,

where V_95%,Body_ is the volume of the body receiving 95% of the prescribed dose. To evaluate the differences between the scheduled and adaptive plans, a paired Wilcoxon signed-rank test was performed. A Bonferroni corrected significance level of 0.4% was used (5%/12). To assess the magnitude of interfraction bladder variability, we determined the bladder volume on the pCTs and CBCT1s for all patients.

To evaluate the automatic target delineation, manual corrections of the GTV delineation during the online sessions (GTV_clin_) were first geometrically assessed. This was done by comparing GTV_clin_ with the AI-supported automatically propagated GTV delineations (GTV_AI_). GTV_AI_ was generated by reproducing the workflow in an online Ethos test environment (Emulator, version 1.1, Varian a Siemens Healthineers Company, USA) with the online acquired CBCTs and the same bladder structure as was used (but without manual corrections to the GTV). The differences between GTV_clin_ and GTV_AI_ were evaluated by calculating the Dice Similarity Coefficient (DSC), Hausdorff Distance (HD) and difference in volume [35, 36]. The difference in volume was also determined with respect to the GTV delineated on the reference plan (GTV_ref_). The next step was to evaluate whether manual corrections of the GTV caused a significant difference in dosimetry. This was done by first exporting the treatment plan from the Emulator (plan_AI_) including GTV_AI_. For each simulated session the same treatment plan was exported as selected during the online session (i.e. adaptive plan or scheduled plan). The V_95%_ of the PTV and CTV and V_95%, out_ as described in the previous section were determined (for PTV_SIB_, CTV_SIB_, PTV_elective_ and CTV_elective_). To compare these parameters with the clinical plan, the clinical delineations (including GTV_clin_) were first propagated to the dose distribution of plan_AI_. The same parameters, i.e. V_95%_ of the PTV and CTV and V_95%, out_, were then determined for these clinical structures. To leave out the possible effect of the learning curve on manual corrections, the training group of the first 5 patients was excluded from the evaluation of the GTV propagation.

The on-couch time was defined as the time from image acquisition (CBCT1) until the end of RT (excluding time spend on patient setup and acquisition of CBCT3). The session time, defined by the time between the patient entering and leaving the treatment room, was manually registered by the RTTs. The duration of different steps in the adaptive procedure was extracted from file records: Patient setup & CBCT1, AI supported structure set propagation & evaluation, Plan reoptimization, Plan evaluation & CBCT2, Position verification, RT & CBCT3 and Patient leaving (see also Fig. 1). The total session time for the training group was compared with the steady group to determine the presence of a learning curve. The comparison was tested for statistical significance by performing a Mann-Whitney test.

Fiducial markers were visually used in the online workflow for delineation of the GTV. We examined the role of these fiducial markers during the structure-guided deformable registration. The position of these fiducial markers on the sCT with respect to the position on the daily CBCT1 was determined. The evaluation was done by first manually delineating the fiducial markers on the pCT, which were subsequently used as input for the simulated sessions in the Emulator. The same steps of the oART workflow as described in the previous section were performed. The marker delineations propagated by the system to the CBCT (Marker_auto_) were compared with manually delineated markers on the CBCT, i.e. Marker_man_ (in the clinical workflow it is these visible markers that are used to determine the location of the tumor by the RTT and physician). The Euclidean Distance between the center of mass of both marker delineations (ΔCoM) were compared (Marker_auto_ vs. Marker_man_). We assumed that an evaluation on the whole data set would not be necessary. Therefore, a representative sample of 40 sessions was created for two groups: sessions where the GTV was manually corrected and sessions where the proposed GTV was accepted (each group half of the sample size). The selection was done by randomly selecting one session per week for all patients in the two different groups. To compare these two groups, a Mann-Whitney test was performed. As also described in the previous section, the training group was excluded here.

## Results

In three patients the patient specific PTV was adjusted based on the early post-treatment evaluations. These adjustments were made after session three for the first patient and after session five and six for the other patients (see also Additional file 1 for more detail). On the post-treatment CBCT (CBCT3), it was observed that the bladder was inside the PTV in 90% of all sessions for all patients (for the initial and interfraction bladder volume see Additional file 4).

### Plan quality and consistency

**Fig. 2:**
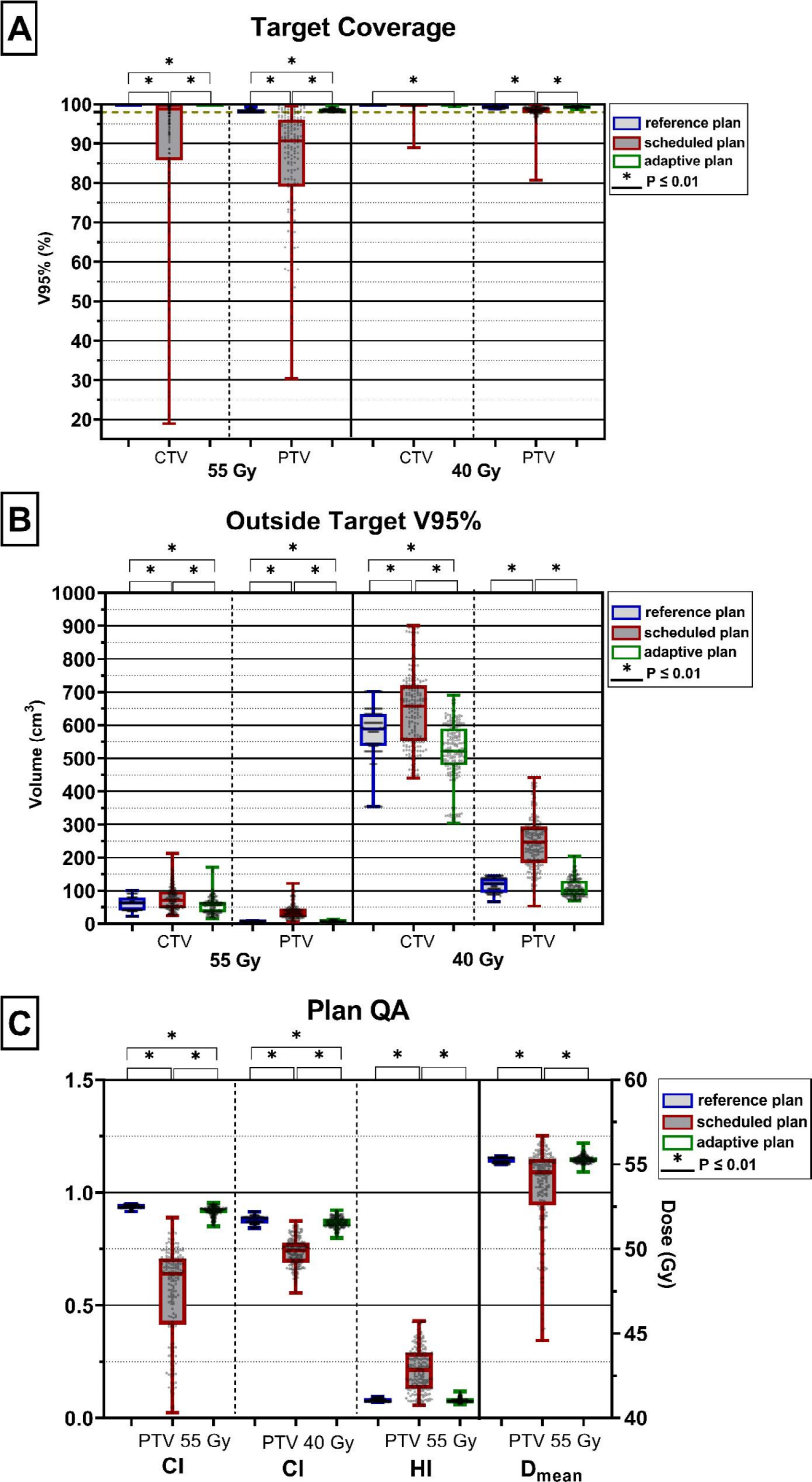
Comparison between the reference plan, scheduled plan and adaptive plan of all sessions from all 15 patients (n = 300 sessions). The boxplots represent the 1st and 3rd quartile, the line inside the median and the whiskers represent the range. **A**) Target coverage of the three plans from left to right: the CTV_SIB_, PTV_SIB_, CTV_elective_ and PTV_elective_. The dotted green line represents the clinical goal. **B**) Volume of external tissue outside the CTV_SIB_ and PTV_SIB_ receiving a dose of 55 Gy and outside the CTV_elective_ and PTV_elective_ receiving a dose of 40 Gy. **C**) The median CI for both the boost and elective PTVs (left axis), the HI for the boost PTV (left axis) and mean dose for the boost area (right axis)

The adaptive plan was selected in 99.7% of the sessions and the scheduled plan was selected once. All 300 adaptive plans met the requirement of the CTV and PTV coverage (V_95%_>98%) for both the boost (55 Gy) and elective (40 Gy) volume (Fig. 2A). For the scheduled plans this requirement was met by 49% and 96% of the treatment plans for CTV_SIB_ and CTV_elective_, respectively. For the PTV_SIB_ this was achieved by 8% and for the PTV_elective_ this was 63% for the scheduled plans. Even though the adaptive plans showed a significant different target coverage compared to the reference treatment plans, it was not a clinically relevant difference as the median target coverage and range were still within the clinical requirements (Fig. 2A). For the scheduled plans, the median target coverage was lower and the range was higher meaning there was less consistency when considering the target coverage. Furthermore, significantly (p < 0.001) less external tissue received high dose (40 and 55 Gy) with the adaptive plans compared to the scheduled and reference plans. An exception was the healthy tissue outside the PTV_elective_ of the adaptive plans, which was comparable to the reference plan (Fig. 2B). The adaptive plans outperformed the scheduled plans with respect to the CI (for more details see also Additional file 5), the HI and D_mean_. Moreover, compared to the scheduled plans, the adaptive plans were closer to the values extracted from the reference plans and showed less variation, i.e. more consistency (Fig. 2C).

### Accuracy and target propagation

**Fig. 3:**
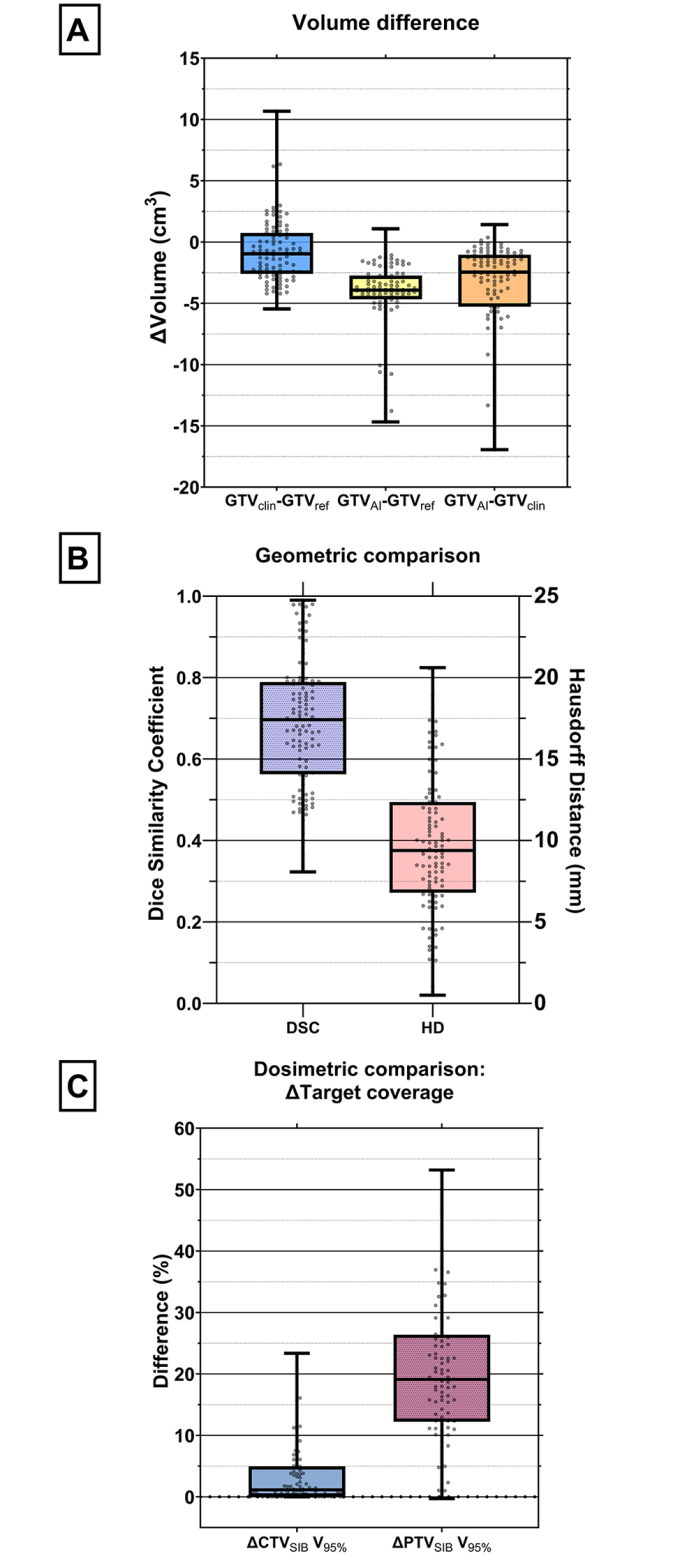
Evaluation of the GTV contour propagation by the Ethos software in sessions where the GTV was manually adjusted (N = 10 patients, n = 134 sessions). The boxplots represent the 1st and 3rd quartile, the line inside the median and the whiskers represent the range. **A**) Difference between the volume of GTV_clin_, GTV_ref_ and GTV_AI_. **B**) The Dice similarity coefficient and Hausdorff distance (mm) between the GTV_clin_ and GTV_AI_. **C**) Difference in target coverage (volume receiving minimum of 95% of the prescribed dose of 55 Gy) between the CTV and PTV of GTV_AI_ (i.e. generated by the Ethos software) and the CTV and PTV of GTV_clin_ (i.e. manually corrected), respectively

Manual adjustment of the GTV was performed in 67% of the sessions of all patients. For the training group (patient 1–5) manual correction of the GTV was done in 80% of the sessions and reduced to 60% for the subsequent 10 patients, i.e. the steady group. 96% of the corrections were performed to significantly (p < 0.001) enlarge the GTV_AI_ delineations with a median volume of 2.5 cm^3^ (Fig. 3a and Additional file 6). The median volume of the resulting GTV_clin_ was 0.9 cm^3^ smaller compared to the reference GTV (p < 0.001). GTV_AI_ was 3.9 cm^3^ smaller than the median volume of GTV_ref_ (p < 0.001). Comparing GTV_clin_ with GTV_AI_ from sessions where manual GTV corrections were performed resulted in a median Dice Similarity Coefficient of 0.70 and a median Hausdorff Distance of 9 mm (Fig. 3b). Performing the GTV corrections, i.e. GTV_clin_, resulted in a significant (p < 0.001) median increase in target coverage of 1% for the V_95%_ of the CTV_SIB_ compared to that same parameter for the GTV_AI_ (Fig. 3c). Manual adjustments of the GTV never resulted in a decrease of the CTV_SIB_ coverage. For the PTV_SIB_, manual corrections of the GTV caused a significant (p < 0.001) increase in target coverage of 19%.

### On-couch time and duration of oART

**Fig. 4:**
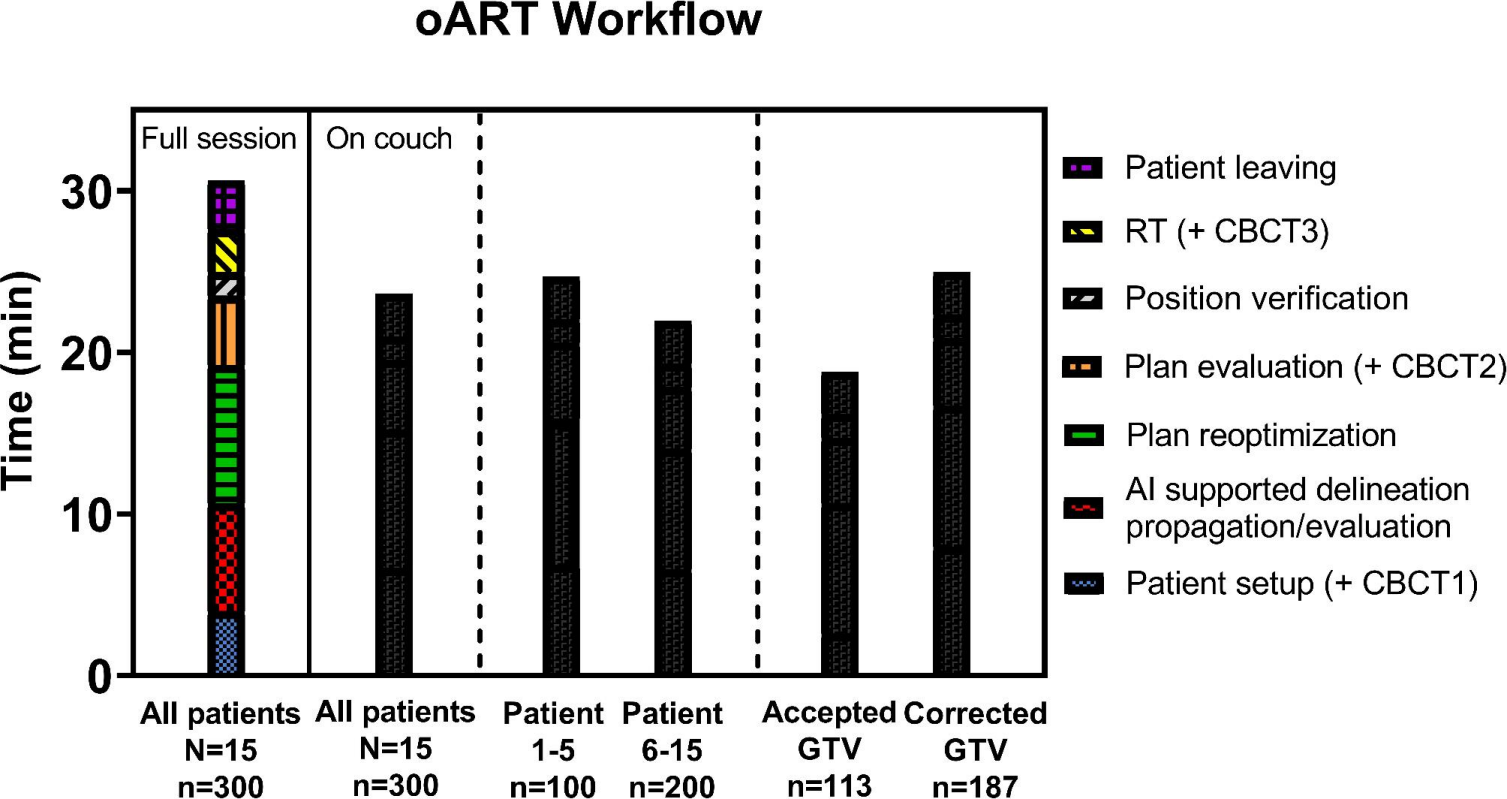
Median session time with the median duration of each step in the oART workflow and median on-couch time for: all patients (N = 15), first five patients, subsequent ten patients, sessions where GTV_AI_ was accepted and sessions where the GTV_AI_ was corrected (N = number of patients and n = number of sessions)

The median session time was 30 min (range: 19–56) for all patients and consisted for about a third of reoptimization of the treatment plan (Fig. 4). Patients setup and acquiring CBCT1 took a median of 4 min, for propagation and evaluation of the structure set this was 7 min and reoptimization of the treatment plan consisted of a median duration of 8 min. For plan evaluation a median of 4 min was needed, for position verification 1–2 min and for dose delivery 2–3 min. The median on-couch time was 22 min for the steady group (range: 14–51) and reduced significantly compared to the training group (p < 0.001). The steady group also showed a shortened plan evaluation time of 1.5 min (see also Additional file 7). Considering sessions in which the GTV delineation was corrected, the AI supported delineation propagation/evaluation reduced with 1.5 min for the steady group in comparison to the training group. For the training group the median on-couch time was 26 min and for the total patient group the median on-couch time was 23 min. Manual correction of the GTV resulted in a median addition of 5 min to the session time compared to session where GTV_AI_ was accepted.

### Fiducial markers

**Fig. 5:**
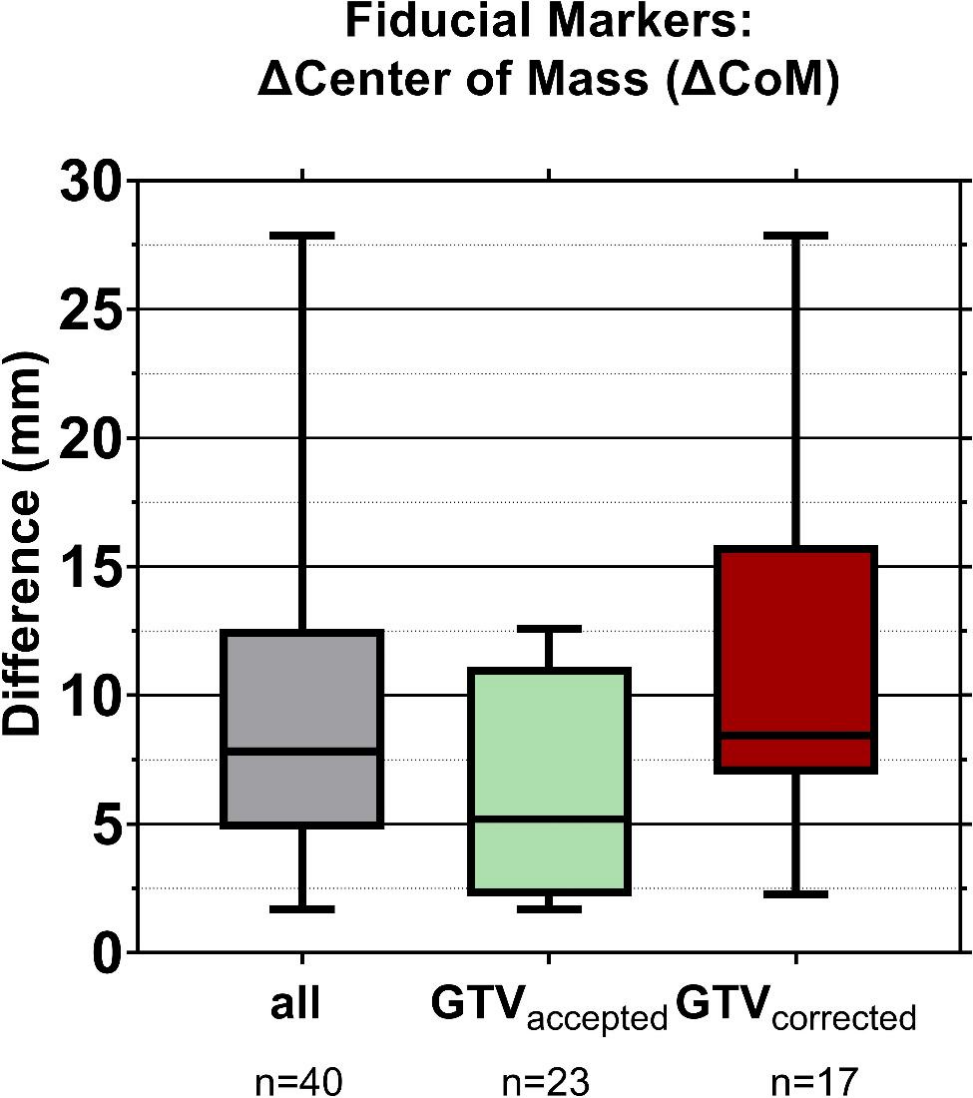
Distance between the center of mass of marker contours propagated by the Ethos software, i.e. Marker_auto_ and manually delineated, i.e. Marker_man_ (N = 10 patients; n = number of sessions). The boxplots represent the 1st and 3rd quartile, the line inside the median and the whiskers represent the range. “GTV corrected” represents the sessions where the GTV was online adjusted. “GTV accepted” represents the sessions where the propagated GTV was accepted. No significant difference was found between these two groups (p < 0.001)

Fiducial markers used as aid during online target evaluation were visible and could be distinguished on the CBCT (Additional file 8). For the representative sample of 40 sessions the center of mass position, CoM, of Marker_auto_ (the fiducial marker delineation propagated by the Ethos system) differed with a median of 7.8 mm from the CoM position given by Marker_man_, i.e. the manually delineated marker (Fig. 5). The median ∆CoM of sessions where GTV_AI_ was immediately accepted was 2.6 mm lower than for the sessions where manual GTV adjustment was performed.

## Discussion

In this study we are the first to demonstrate the feasibility of the CBCT-guided oART workflow for bladder cancer using a focal boost and fiducial markers. With the adaptive procedure online treatment plans with excellent conformity were generated with a median on-couch time, after a training period, of 22 min. These adaptive plans always met the clinical requirements considering the target coverage and reduced the high dose to healthy tissue outside the boost and elective targets compared to the reference plan.

The superior target coverage, healthy tissue sparing and plan quality of the adaptive plans compared to the scheduled plans demonstrate the benefit of implementing online reoptimization of the treatment plan. These aspects could be maintained while allowing for smaller PTVs which is in line with previous studies [19, 21, 22, 28]. The target coverage of the clinically used online plans always met the clinical requirement which is also observed by previous CBCT- and MR-guided oART studies [18, 28]. In terms of the HI and CI, the plan quality was also comparable with a previous CBCT-guided oART study for bladder cancer [28]. These similarities between our CI values (using VMAT) and the CI values reported by Åström et al. (using IMRT), imply that the delivery method does not need to have an effect on the conformity [28]. Foroudi et al. published about the differences between the delivery methods and found no significant difference in CI between IMRT and VMAT for bladder cancer patients [37]. However, for MR-guided oART the conformity, CI_RTOG_, is reported to be around 2.5 indicating considerable dose to healthy tissue [18]. In our study the conversion of the CI to CI_RTOG_ was between 1.0 and 1.3 for all adaptive plans, showing an improved conformity (with the ideal CI_RTOG_ value equal to 1). In view of the observed similarity of IMRT and VMAT, as mentioned above [37] it seems more likely that this is due to larger treatment times (and corresponding larger margins to deal with intrafraction motion). Furthermore, a previous study on employing LoP for the treatment of bladder cancer, including a SIB, reported an even higher value for CI_RTOG_ of 3.5 [38], which might be explained by interfraction variability of the bladder shape as compared to the available library plans. These findings suggests the potential gain of dose reduction to healthy tissue by reoptimized CBCT-guided oART for bladder cancer as compared to MR-guided oART and LoP.

When no manual corrections were needed, the session time was reduced with 5 min compared to sessions where the GTV was adjusted. The GTV volume appeared to be smaller when delineated by the Ethos software as compared to the clinically manually adapted GTV. The geometric differences between GTV_clin_ and GTV_AI_ were large enough to result in significant dosimetric differences, i.e. lower dose in the tumor. The manual adjustments of the GTV performed in 6 out of 10 sessions resulted in longer session times. More accurate GTV propagation by the software is needed to allow for a shorter workflow and thus less intrafraction bladder filling and potentially smaller PTV margins. The effect of interobserver variation during correction of manual corrections was not considered in this study.

A detailed time schedule of the oART workflow was analyzed giving departments a more detailed insight of what to expect during clinical implementation. The on-couch time was about 10 min lower than reported by Hunt et al. for MR-guided oART for bladder cancer [18]. Plan reoptimization took about 2 min longer in our study and the dose delivery time was 6 min shorter. Their findings show that the recontouring part of the workflow took 7 min which is the same as the time for our total structure set propagation and performance of manual correction. Reducing the need for manual correction would result in a structure set delineation time of 2 min as shown by the fully automatic sessions. The on-couch time would be 18% shorter, meaning a reduction of the total session time of 5 min. Therefore, improving the software for accurate GTV propagation would be beneficial for bladder treatments.

Compared to a previous CBCT-guided whole bladder oART study without a focal boost to the tumor, the review of the automatically generated structure set was about 2 min shorter in our case [28]. The plan reoptimization and selection took approximately 6 min longer resulting in a longer adaptive procedure of 4 min [28]. The main difference between these studies, including a SIB (thus two dose levels) and employing VMAT instead of IMRT, apparently leads to a longer reoptimization time in our case. However, VMAT has shown to deliver treatment within a shorter time compared to IMRT [37]. Compared to Library of Plans, as reported by a study including a SIB, the on-couch time was about 10 min longer in our study [38].

The on-couch time decreased for patient 6 to 15, as compared to the first 5 patients. This is indicative of a learning curve caused by experience with evaluating the treatment plan. The learning curve regarding the duration of the manual corrections yielded an overall speed up of the work flow. This might be due to the staff’s increasing skill in applying the manual corrections. Additionally, during the pilot phase manual corrections could be made that afterwards would not be considered clinically relevant. As the staff gaining experience, the frequency of performing manual corrections decreased. As 96% of the corrections were made to increase the GTV, we do not expect this to have an effect on the target coverage of the first 5 patients compared to the latter 10 patients, but we do expect it to affect the volume of healthy tissue receiving high dose (i.e. 40/55 Gy). The decrease in frequency of manual corrections also gives another explanation for the reduction in session time. Longer session times would result in more intrafraction bladder filling necessitating larger PTV margins. Nevertheless, our study shows that the bladder was included by the PTV at CBCT3 in 90% of the sessions. A limitation of this study is the assumption of linear bladder filling with a fixed filling rate between the 2 pCTs. Additionally, the different session times are currently not taken into account. However, the CBCT acquired after dose delivery allows for monitoring and gives the possibility to modify the PTV margins in between treatment fractions.

To be superior to LoP with respect to the irradiated volume of healthy tissue, an MR-guided study suggested that the session time should be around 15 min [39]. Another preclinical study reported the requirement of a PTV margin of 5 mm [21]. These requirements are currently not met by the oART workflow on the Ethos. It should be noted that these requirements were for whole bladder treatments without a SIB and requirements for treatments including a SIB are to our knowledge currently lacking. An advantage of the oART workflow over LoP, is taking into account the OARs [40]. This is a potential benefit especially for patients with the small bowel close to the tumor. Besides the difference in conformity mentioned earlier, this also illustrates the potential for the reoptimization oART workflow to reduce gastrointestinal toxicity. Furthermore, during LoP it might occur that no suitable treatment plan fits the daily anatomy, which is not the case for oART [41].

To allow for accurate boost dose to the tumor, fiducial markers were used for tumor localization. The markers could be clearly distinguished on the online CBCT images and was an essential part of the target evaluation and adaptation process. However, the system was not able to localize the markers accurately enough on the daily anatomy with aid of the structure-guided deformable registration. Sessions where manual correction of the GTV was performed showed a larger displacement of the markers. Allowing the structure-guided deformable registration to account for fiducial markers, as they are used for tumor localization, might increase the accuracy and lower the treatment time when using a focal boost.

A limitation of our study was that we evaluated the dose on CBCT1. An evaluation of the dose on CBCT2 or CBCT3 would probably better reflect the actually delivered dose. In our paper we choose to focus on the data that is used for the actual clinical decision making, as reflected on CBCT1. This is in line with earlier studies with a LoP, also using the initial (and only) CBCT for dose evaluation [38]. Future investigation should reveal if there is significant difference between the dose evaluated on CBCT1 and CBCT2 or CBCT3 which is not unlikely due the time between the scans. Additionally, using a larger number of patients for the study would make the data more representative considering the generalizability.

## Conclusion

We have shown the clinical feasibility of delivering a focal boost with aid of fiducial markers during daily online adaptive radiotherapy for muscle-invasive bladder cancer. Employing an AI-driven CBCT-guided RT technique yields consistent plan quality for all treatments, comparable to the pretreatment reference plan with a median on-couch time of 22 min.

### Electronic supplementary material

Below is the link to the electronic supplementary material.

Supplementary Material 1. Additional file 1 (.pdf) : Patient characteristics including sex, age, tumor stage and other.

Supplementary Material 2. Additional file 2 (.pdf) : Representation of a planning CT used to calculate the reference plan.

Supplementary Material 3. Additional file 3 (.pdf) : Representation of the template with prioritized planning directives used to constraint treatment plans. The exact values of the clinical goals were patient-specific.

Supplementary Material 4. Additional file 4 (.pdf) : The initial bladder volume from the pCT and the interfraction bladder volume from CBCT1.

Supplementary Material 5. Additional file 5 (.pdf) : The conformity index (CI_RTOG_ = V_95%_ / PTV volume) of the adaptive plans for the tumor and elective area (N = 15 patients = 300 sessions). Different ways of calculating the CI are used in literature. To be able to compare our work with previous studies we added the conformity index as proposed by the Radiation Therapy Oncology Group (RTOG) [34].

Supplementary Material 6. Additional file 6 (.pdf) : A representation of the automatic propagated GTV (GTV_AI_), the manually corrected GTV delineation on the online CBCT (GTV_clin_) and the reference GTV on the planning CT.

Supplementary Material 7. Additional file 7 (.pdf) : Comparison between the training group (P1-5) and steady group (P6-15) considering the duration of different steps from the oART workflow. The number of sessions is indicated by n.

Supplementary Material 8. Additional file 8 (.pdf) : Representation of a GTV delineation (yellow) on an online CBCT (from up to down: axial, sagittal and coronal). Fiducial markers (white spots) indicated with red arrows are used as aid for the delineation.

## Data Availability

The datasets used and/or analyzed during the current study are available from the corresponding author on reasonable request.

## Abbreviations

AI: Artificial intelligence
CBCT: Cone Beam CT
CI: Conformity index
CoM: Center of mass
CT: Computed tomography
CTV: Clinical target volume
DSC: Dice Similarity Coefficient
FFF: Flattening-Filter-Free
Gy: Gray
GTV: Gross tumor volume
HD: Hausdorff Distance
HI: Homogeneity index
LoP: Library of plans
MRI: Magnetic resonance imaging
MV: Megavolt
OAR: Organ-at-risk
oART: Online adaptive radiotherapy
pCT: Planning computed tomography
PTV: Planning target volume
QA: Quality assurance
RT: Radiotherapy
RTT: Radiation therapist
SIB: Simultaneously integrated boost
sCT: Synthetic computed tomography
TPS: Treatment planning system
TURBT: Transurethral resection of bladder tumor
TV: Target volume
V_95%_: Volume receiving a minimum of 95% of the prescribed dose
VMAT: Volumetric modulated arc therapy
