# Supplementary material for: Online adaptive radiotherapy for bladder cancer using a simultaneous integrated boost and fiducial markers
Source: Radiat Oncol. 2023 Oct 6;18:165. doi: 10.1186/s13014-023-02348-8 (PMC10557331; doi:10.1186/s13014-023-02348-8)
Supplement: Supplementary file 1 — Supplementary Material 1. Additional file 1 (.pdf) : Patient characteristics including sex, age, tumor stage and other. [file 13014_2023_2348_MOESM1_ESM.pdf]

Patient characteristics

Patient	Sex	Age	Tumor stage	Other
**1**	M	79	(cT2N0M0)G3	
**2**	M	65	(cT2N0M0)G3	
**3**	M	70	(cT3N0M0)G3	
**4**	M	57	(cT3N0M0)G3	
**5**	M	66	(pT1N0M0)G3	
**6**	M	63	(cT3N0M0)G3	PTV margins adjusted (ant: 1.7→1.0 cm, other directions stayed 0.7 cm)
**7**	F	71	(cT2N0M0)G3	
**8**	F	66	(cT2N0M0)G3	
**9**	M	79	(cT2N0M0)G3	PTV margins adjusted (ant: 3.0→0.7 cm, post: 0.7→1.5 cm, sup: 2.5→0.7 cm, inf: 0.7→1.5 cm, other directions stayed 0.7 cm)
**10**	M	62	(cT2N0M0)G3	
**11**	F	61	(pT2aN0M0)G3	
**12**	M	82	(cT2N0M0)G3	
**13**	F	78	(cT2N0M0)G3	PTV margins adjusted (sup: 2.0→1.2 cm, other directions stayed 0.7 cm)
**14**	M	49	(cT2N0M0)G3	
**15**	M	66	(cT2N1M0)G3	

Additional file 1 : Patient characteristics including sex, age, tumor stage and other.
