# Supplementary material for: Online adaptive radiotherapy for bladder cancer using a simultaneous integrated boost and fiducial markers
Source: Radiat Oncol. 2023 Oct 6;18:165. doi: 10.1186/s13014-023-02348-8 (PMC10557331; doi:10.1186/s13014-023-02348-8)
Supplement: Supplementary file 2 — Supplementary Material 2. Additional file 2 (.pdf) : Representation of a planning CT used to calculate the reference plan. [file 13014_2023_2348_MOESM2_ESM.pdf]

Planning CT

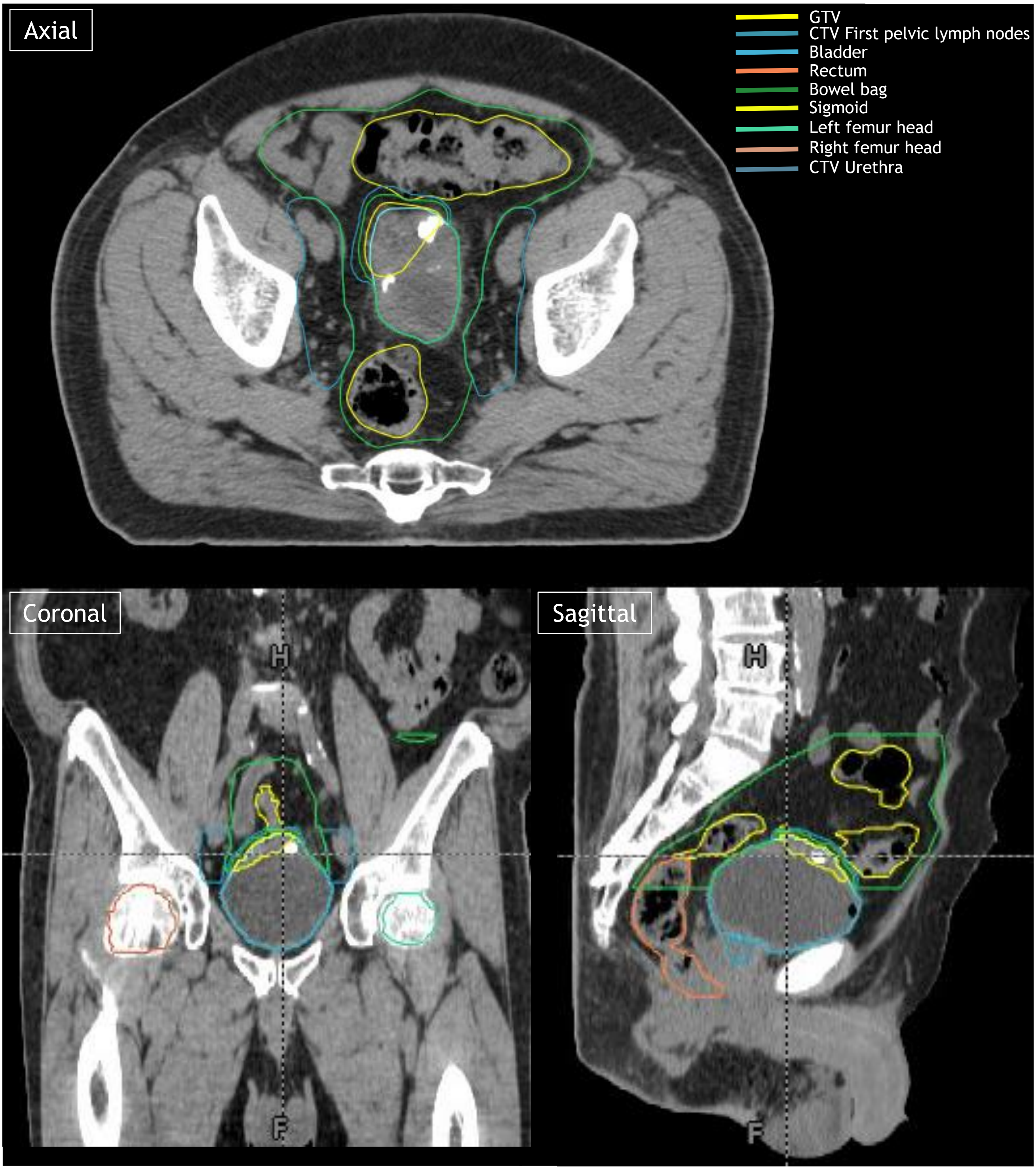

Additional file 2 : Representation of a planning CT used to calculate the reference plan.
