# Supplementary material for: Online adaptive radiotherapy for bladder cancer using a simultaneous integrated boost and fiducial markers
Source: Radiat Oncol. 2023 Oct 6;18:165. doi: 10.1186/s13014-023-02348-8 (PMC10557331; doi:10.1186/s13014-023-02348-8)
Supplement: Supplementary file 3 — Supplementary Material 3. Additional file 3 (.pdf) : Representation of the template with prioritized planning directives used to constraint treatment plans. The exact values of the clinical goals were patient-specific. [file 13014_2023_2348_MOESM3_ESM.pdf]

Clinical goals

Structure	Goal	Priority
GTV_{boost}	V101.5% > 100%	1
$PTV_{elective}$	V95 ≥ 98%	1
PTV_{boost}	V95 ≥ 98%	1
	V58.85 Gy ≤ 2 cm^3	1
	Dmax < 110%	1
	Dmean > 100%	1
$PTV_{elective}$ - PTV_{boost}	V107% ≤ 1 cm^3	1
$PTV_{elective}$ – (PTV_{boost} + 0.7 cm)	V107% ≤ 1 cm^3	2
Bowel small	V55 Gy ≤ 3 cm^3	2
Rectum	V55 Gy ≤ 12 cm^3	2
	V50 Gy ≤ 15%	2
Rectum – $PTV_{elective}$	V40 Gy ≤ 1 cm^3	2
	V30 Gy ≤ 6 cm^3	2
	V15 Gy ≤ 19 cm^3	3
Sigmoid	V55 Gy ≤ 12 cm^3	2
Bowel bag – $PTV_{elective}$	V40 Gy ≤ 25 cm^3	2
	V32.5 Gy ≤ 60 cm^3	2
	V25 Gy ≤ 150 cm^3	2
	V15 Gy ≤ 300 cm^3	3
Femur heads	D2 cm^3 ≤ 42 Gy	3

Additional file 3 : Representation of the template with prioritized planning directives used to constraint treatment plans. The exact values of the clinical goals were patient-specific.
