# Supplementary material for: Online adaptive radiotherapy for bladder cancer using a simultaneous integrated boost and fiducial markers
Source: Radiat Oncol. 2023 Oct 6;18:165. doi: 10.1186/s13014-023-02348-8 (PMC10557331; doi:10.1186/s13014-023-02348-8)
Supplement: Supplementary file 4 — Supplementary Material 4. Additional file 4 (.pdf) : The initial bladder volume from the pCT and the interfraction bladder volume from CBCT1. [file 13014_2023_2348_MOESM4_ESM.pdf]

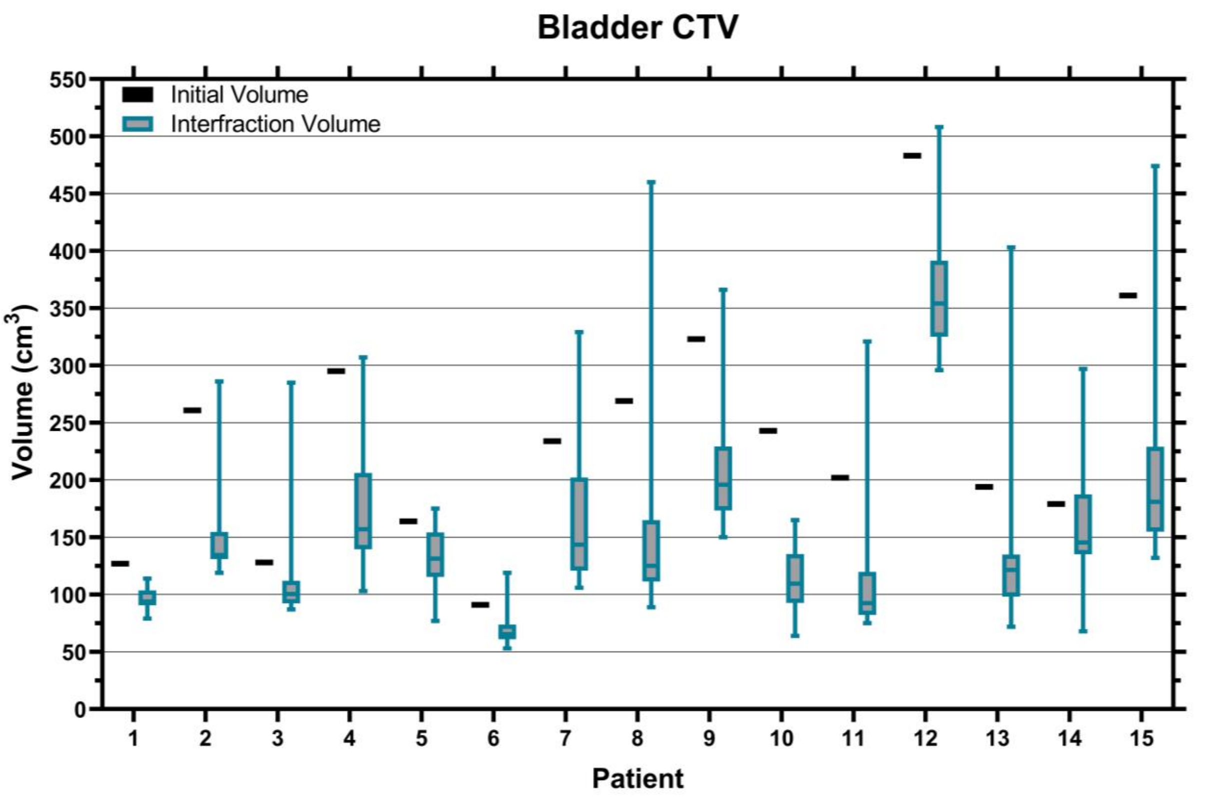

Additional file 4 : The initial bladder volume on the planning CT and the interfraction bladder volume on CBCT1 (20 fractions per patient).
