# Supplementary material for: Online adaptive radiotherapy for bladder cancer using a simultaneous integrated boost and fiducial markers
Source: Radiat Oncol. 2023 Oct 6;18:165. doi: 10.1186/s13014-023-02348-8 (PMC10557331; doi:10.1186/s13014-023-02348-8)
Supplement: Supplementary file 5 — Supplementary Material 5. Additional file 5 (.pdf) : The conformity index (CIRTOG = V95% / PTV volume) of the adaptive plans for the tumor and elective area (N = 15 patients = 300 sessions). Different ways of calculating the CI are used in literature. To be able to compare our work with previous studies we added the conformity index as proposed by the Radiation Therapy Oncology Group (RTOG) [34]. [file 13014_2023_2348_MOESM5_ESM.pdf]

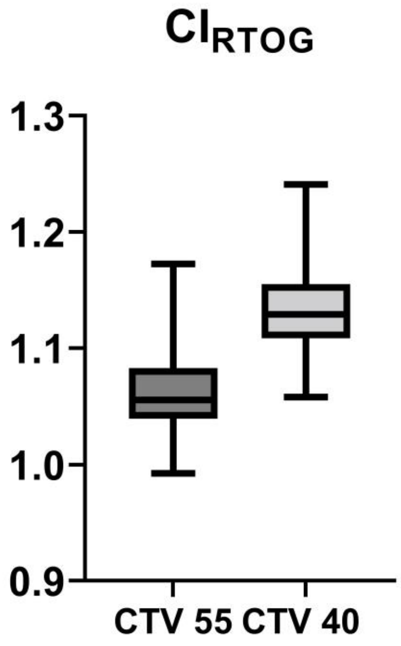

Additional file 5 : The conformity index (CI_{RTOG} = V95% / PTV volume) of the adaptive plans for the tumor and elective area (N = 15 patients = 300 sessions). Different ways of calculating the CI are used in literature. To be able to compare our work with previous studies we added the conformity index as proposed by the Radiation Therapy Oncology Group (RTOG) [35] .
