# Supplementary material for: Online adaptive radiotherapy for bladder cancer using a simultaneous integrated boost and fiducial markers
Source: Radiat Oncol. 2023 Oct 6;18:165. doi: 10.1186/s13014-023-02348-8 (PMC10557331; doi:10.1186/s13014-023-02348-8)
Supplement: Supplementary file 6 — Supplementary Material 6. Additional file 6 (.pdf) : A representation of the automatic propagated GTV (GTVAI), the manually corrected GTV delineation on the online CBCT (GTVclin) and the reference GTV on the planning CT. [file 13014_2023_2348_MOESM6_ESM.pdf]

GTV-delineation propagation

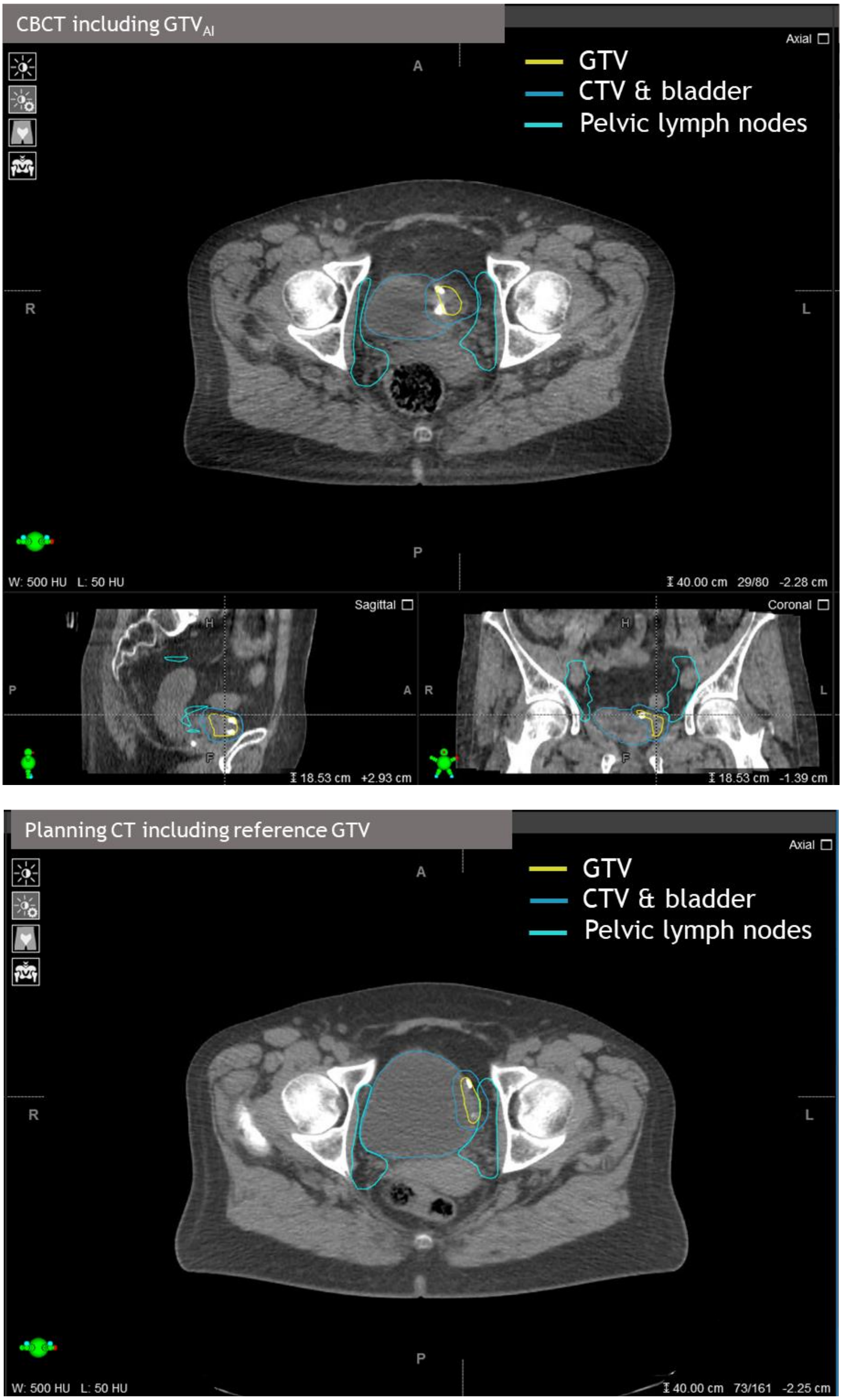

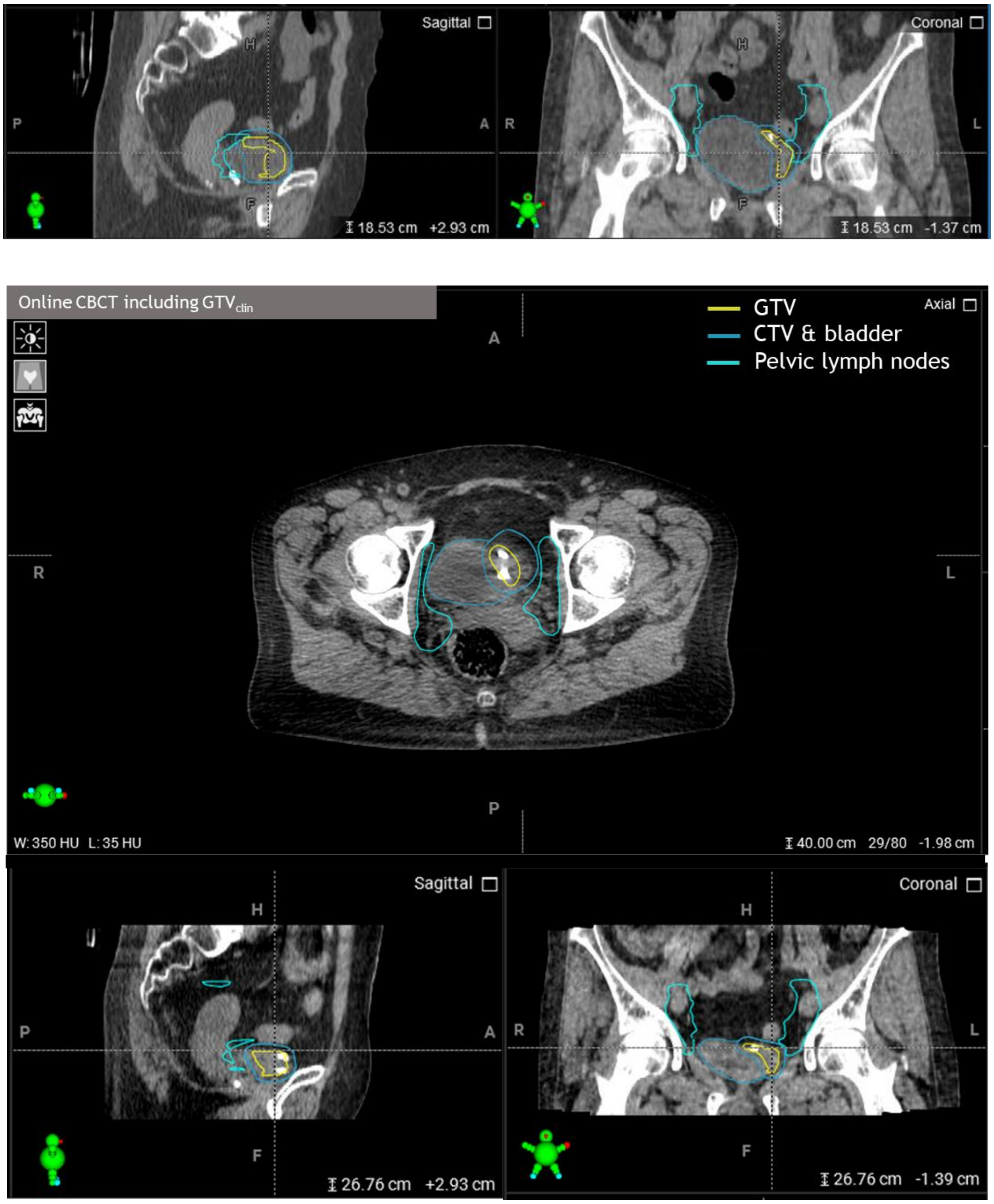

Additional file 6 : A representation of the automatic propagated GTV (GTV_{AI}), the manually corrected GTV delineation on the online CBCT (GTV_{clin}) and the reference GTV on the planning CT.
