# Supplementary material for: Online adaptive radiotherapy for bladder cancer using a simultaneous integrated boost and fiducial markers
Source: Radiat Oncol. 2023 Oct 6;18:165. doi: 10.1186/s13014-023-02348-8 (PMC10557331; doi:10.1186/s13014-023-02348-8)
Supplement: Supplementary file 7 — Supplementary Material 7. Additional file 7 (.pdf) : Comparison between the training group (P1-5) and steady group (P6-15) considering the duration of different steps from the oART workflow. The number of sessions is indicated by n. [file 13014_2023_2348_MOESM7_ESM.pdf]

Comparison between training group and steady group

**Duration** (min)	Training group<br><br>*all sessions*<br><br>*n = 100*	Steady group<br><br>*all sessions*<br><br>*n = 200*	Training group<br><br>*sessions with corrected* GTV_{AI}<br><br>*n = 80*	Steady group<br><br>*sessions with corrected* GTV_{AI}<br><br>*n = 120*	Training group<br><br>*sessions with accepted* GTV_{AI}<br><br>*n = 20*	Steady group<br><br>*sessions with accepted* GTV_{AI}<br><br>*n = 80*
**AI supported delineation propagation/ evaluation**	7	$6\frac{1}{2}$	9	$7\frac{1}{2}$	3	2
**Plan reoptimization**	9	$7\frac{1}{2}$	9	$7\frac{1}{2}$	9	8
**Plan evaluation (+ CBCT2)**	5	4	5	$3\frac{1}{2}$	$4\frac{1}{2}$	4
**Position verification**	$1\frac{1}{2}$	$1\frac{1}{2}$	$1\frac{1}{2}$	$1\frac{1}{2}$	2	$1\frac{1}{2}$
**RT (+ CBCT3)**	$2\frac{1}{2}$	$2\frac{1}{2}$	$2\frac{1}{2}$	$2\frac{1}{2}$	$2\frac{1}{2}$	$2\frac{1}{2}$

Additional file 7 : Comparison between the training group (P1-5) and steady group (P6-15) considering the duration of different steps from the oART workflow. The number of sessions is indicated by n.
