# Supplementary material for: Online adaptive radiotherapy for bladder cancer using a simultaneous integrated boost and fiducial markers
Source: Radiat Oncol. 2023 Oct 6;18:165. doi: 10.1186/s13014-023-02348-8 (PMC10557331; doi:10.1186/s13014-023-02348-8)
Supplement: Supplementary file 8 — Supplementary Material 8. Additional file 8 (.pdf) : Representation of a GTV delineation (yellow) on an online CBCT (from up to down: axial, sagittal and coronal). Fiducial markers (white spots) indicated with red arrows are used as aid for the delineation. [file 13014_2023_2348_MOESM8_ESM.pdf]

Fiducial markers

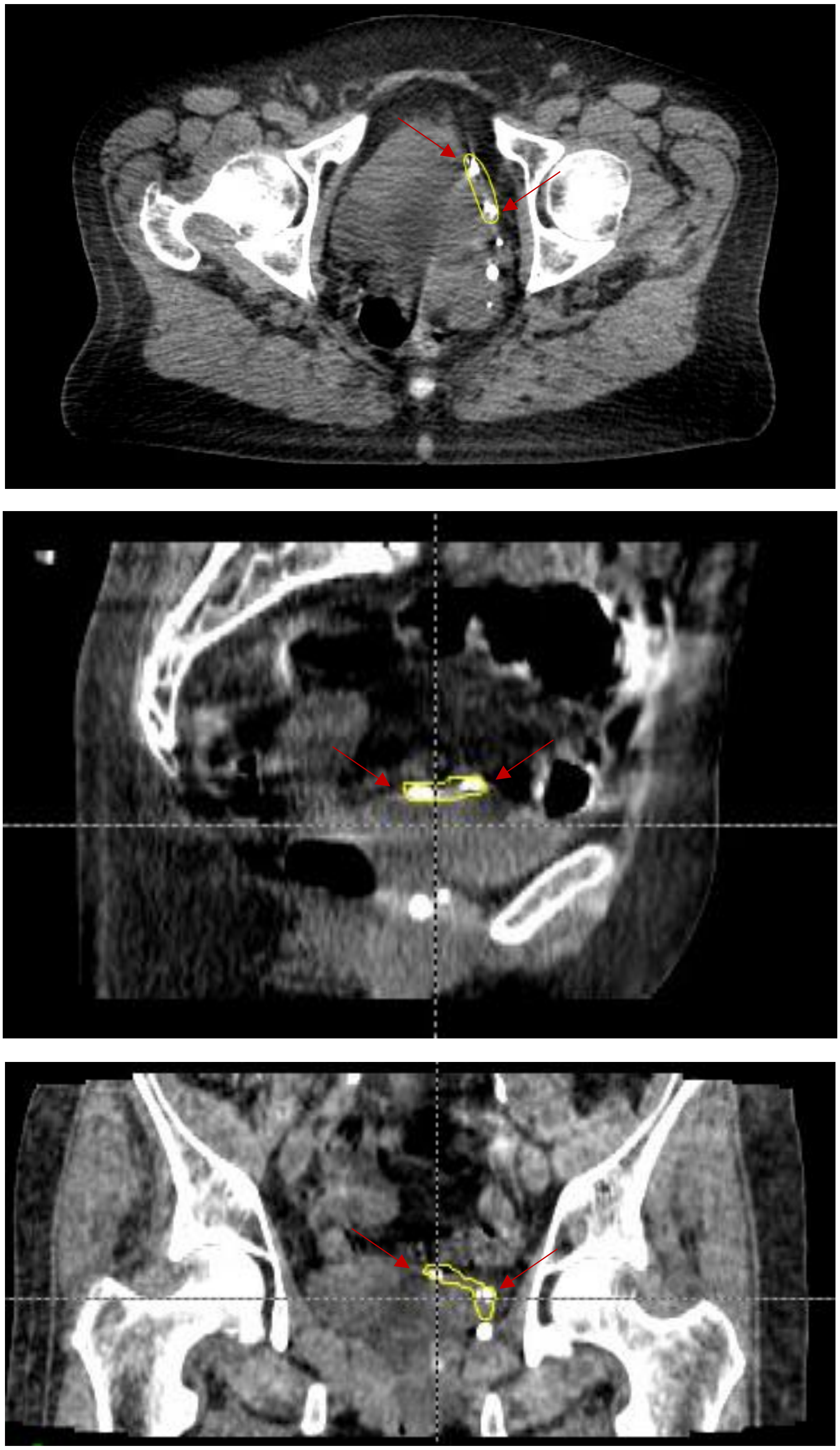

Additional file 8 : Representation of a GTV delineation (yellow) on an online CBCT (from up to down: axial, sagittal and coronal). Fiducial markers (white spots) indicated with red arrows are used as aid for the delineation.
